# Self-management behaviour and support among primary care COPD patients: cross-sectional analysis of data from the Birmingham Chronic Obstructive Pulmonary Disease Cohort

**DOI:** 10.1038/s41533-017-0046-6

**Published:** 2017-07-20

**Authors:** Ainee Khan, Andrew P. Dickens, Peymane Adab, Rachel E. Jordan

**Affiliations:** 1 Public Health, Solihull Metropolitan Borough Council, Solihull, B91 3QB UK; 2 Institute of Applied Health Research, University of Birmingham, Birmingham, B15 2TT UK

## Abstract

Self-management support for chronic obstructive pulmonary disease (COPD) patients is recommended by UK national guidelines, but extent of implementation is unknown. We aimed to describe self-management behaviour and support among COPD patients and explore behaviour associated with having a self-management plan. We undertook cross-sectional analysis of self-reported data from diagnosed COPD patients in the Birmingham COPD Cohort study. Questionnaire items relevant to self-management behaviour, knowledge of COPD, receipt of self-management plans and advice from healthcare professionals were examined. Multiple regression models were used to identify behaviour associated with having a self-management plan. One-thousand seventy-eight participants (676 males, 62.7%, mean age 69.8 (standard deviation 9.0) years) were included. The majority reported taking medications as instructed (940, 94.0%) and receiving annual influenza vaccinations (962, 89.2%). Only 400 (40.4%) participants had self-management plans, 538 (49.9%) reported never having received advice on diet/exercise and 110 (42.7%) current smokers had been offered practical help to stop smoking in the previous year. General knowledge about COPD was moderate (mean total Bristol COPD Knowledge Questionnaire score: 31.5 (standard deviation 10.7); max score 65), corresponding to 48.5% of questions answered correctly. Having a self-management plan was positively associated with self-reported adherence to medication (odds ratio 3.10, 95% confidence interval 1.43 to 6.72), attendance at a training course (odds ratio 2.72, 95% confidence interval 1.81 to 4.12), attendance at a support group (odds ratio 6.28, 95% confidence interval 2.96 to 13.35) and better disease knowledge (mean difference 4.87, 95% confidence interval 3.16 to 6.58). Primary care healthcare professionals should ensure more widespread implementation of individualised self-management plans for all patients and improve the lifestyle advice provided.

## Chronic lung disease: Call for wider implementation of self-management plans

Health professionals should ensure all patients with chronic lung disease receive individualized self-management plans and lifestyle advice. UK national guidelines state that patients with chronic obstructive pulmonary disease (COPD) should receive personalized self-management plans and comprehensive support to help them manage their disease. Ainee Khan and colleagues at the University of Birmingham analyzed patient questionnaire data gathered during the Birmingham COPD Cohort study to explore self-management behavior, receipt of self-management plans and advice, and patient knowledge of COPD. Of 1,078 participants, only 400 had self-management plans, and less than half reported receiving lifestyle advice or support. Those with plans were more likely to adhere to medication, had greater knowledge about COPD and were more likely to attend support groups and training courses. The authors recommend carefully-planned, wider implementation of COPD self-management plans and associated support.

## Introduction

Chronic obstructive pulmonary disease (COPD) is an important long-term condition characterised by persistent decline in airflow^1^ and increasing breathlessness. It affects at least 1.9% of the UK population^2^ and is a costly disease with acute exacerbations being the second leading cause of emergency respiratory hospital admissions in England,^3^ with a similar burden worldwide. Such exacerbations lead to poor prognosis for patients, with reductions in health-related quality of life and increased risk of mortality.^4^

Support to help COPD patients self-manage their condition is recommended by national guidelines to improve their health-related quality of life and reduce avoidable inpatient admissions.^5, 6^ Exact definitions of self-management and the most effective components of interventions to support patients are ongoing debates.^7^ Self-management support programmes should be collaborative between healthcare professionals and patients, to help them acquire skills to understand and manage their medications and exacerbations, adopt healthier behaviours and manage the social-emotional consequences of the disease.^7–11^ Systematic reviews among patients with COPD show that overall, interventions by healthcare professionals to support patient self-management reduce respiratory-related hospital admissions and improve quality of life,^9^ although the content of the interventions are complex and heterogeneous and which components are the most important is still unclear.^12, 13^

Despite limitations in our understanding of which self-management support strategies are the most effective, guidance from the National Institute for Health and Care Excellence (NICE) recommends that all patients with COPD should receive an individualised comprehensive management plan that includes information and educational material about the condition and its management, and that those at risk of exacerbations should be offered advice about how to recognise and respond promptly to the symptoms of an exacerbation.^5, 6^

The literature describing self-management behaviours of COPD patients in the UK and the support they receive is minimal.^14^ In this paper, we describe the current self-management behaviours of patients with COPD in a large primary care cohort in Birmingham, UK. We also describe the support that patients report receiving from their healthcare professionals and whether a collaborative self-management plan is associated with positive self-management behaviours in real life. Our study, therefore, may help in designing and implementing future self-management interventions.

## Results

### Study participants

**Fig. 1:**
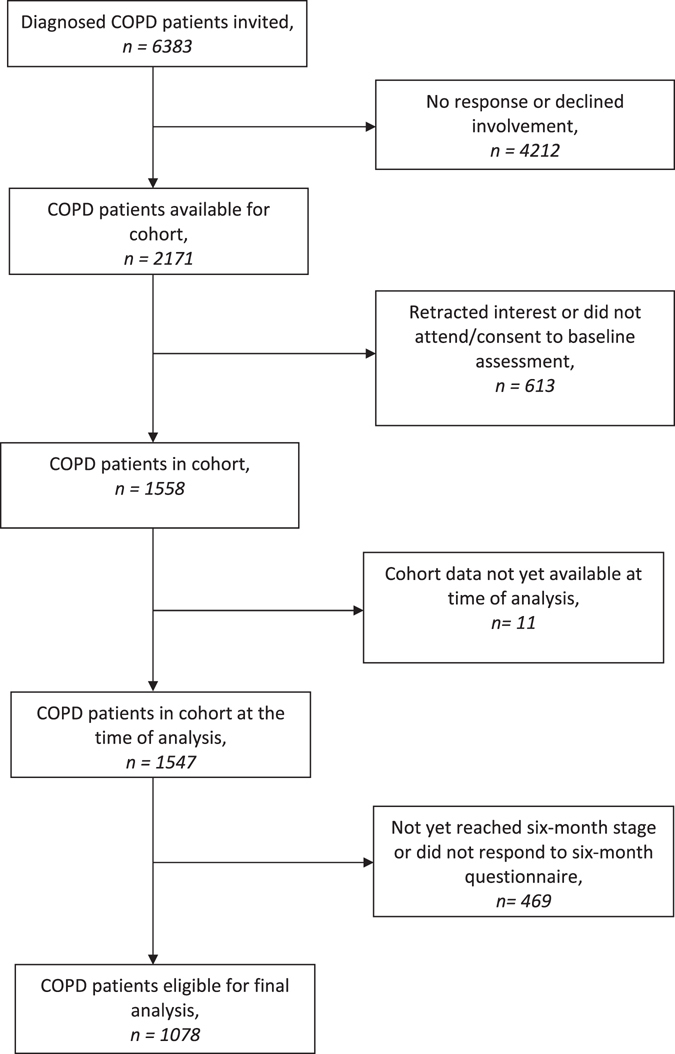
Flow of participants from cohort to sample

At the time of analysis, 1078/1547 participants (69.7%) had returned a valid 6-month questionnaire (Fig. 1).

Of the 1078 respondents, 676 (62.7%) were male, 258 (24.3%) were current smokers and 653 (61.5%) were former smokers (Table 1). The mean age was 69.8 (standard deviation (SD) 9.0) years and they were predominately of White ethnicity (997; 97.6%). The majority were either overweight or obese (786; 72.9%) and had no formal educational qualifications (597; 60.4%). A small proportion of the respondents were employed (154; 14.4%), and over a quarter (302; 29.1%) were living alone.

**Table 1:** Baseline characteristics: all those eligible for analysis in cohort and respondents to 6-month questionnaire

Characteristic	All those diagnosed with COPD (*n* = 1547)	Diagnosed with COPD and responded to 6-month questionnaire (*n* = 1078)
	*N* (%)	*N* (%)
Age (years)		
Mean (SD)	69.2 (9.4)	69.8 (9.0)
Sex		
Male	952 (61.5%)	676 (62.7%)
Female	595 (38.5%)	402 (37.3%)
Smoking status		
Current-smoker	402 (26.6%)	258 (24.3%)
Former-smoker	867 (57.4%)	653 (61.5%)
Never-smoker	242 (16.0%)	151 (14.2%)
Body mass index		
Underweight < 18.5	33 (2.1%)	23 (2.1%)
Normal 18.5–25	387 (25.0%)	269 (25.0%)
Overweight 25–30	566 (36.6%)	403 (37.4%)
Obese > 30	561 (36.3%)	383 (35.5%)
Ethnicity		
White	1377 (97.0%)	997 (97.6%)
Any other	43 (3.0%)	25 (2.4%)
Education		
No formal qualification	838 (61.2%)	597 (60.4%)
GCSE/A-level	288 (21.0%)	202 (20.4%)
Degree	93 (6.8%)	74 (7.5%)
Other qualification	150 (11.0%)	115 (11.6%)
Employment status		
Paid/self-employed	245 (16.0%)	154 (14.4%)
Not working	1286 (84.0%)	919 (85.6%)
Marital status		
Married/civil partner	805 (60.2%)	605 (62.1%)
Never married/civil partner	101 (7.6%)	72 (7.4%)
Widowed	230 (17.2%)	166 (17.0%)
Divorced	177 (13.2%)	116 (11.9%)
Separated	24 (1.8%)	16 (1.6%)
GOLD stage^a^		
FEV1/FVC ≥ 0.7	200 (13.7%)	128 (12.3%)
1 (FEV_1_ ≥ 80% predicted)	308 (21.1%)	225 (21.6%)
2 (50% ≤ FEV_1_ < 80% predicted)	652 (44.7%)	476 (45.8%)
3 (30% ≤ FEV_1_ < 50% predicted)	253 (17.4%)	179 (17.2%)
4 (FEV_1_ < 30% predicted)	45 (3.1%)	32 (3.1%)
Self-reported		
Co-morbidities at baseline		
Cancer	183 (12.3%)	134 (12.7%)
Diabetes	227 (15.2%)	151 (14.3%)
Hypertension	669 (44.8%)	481 (45.4%)
Coronary heart disease	226 (15.1%)	159 (15.0%)
Heart failure	110 (7.4%)	75 (7.1%)
Asthma	611 (40.9%)	408 (38.5%)
Depression	278 (18.6%)	174 (16.4%)
MRC dyspnoea		
Grade 1–2	549 (38.1)	391 (37.1)
Grade 3–5	893 (61.9)	662 (62.9)
CAT		
Score; mean (SD)	20.0 (8.8)	19.3 (8.6)
Living situation		
Alone	418 (29.0%)	302 (29.1%)
Not alone	1021 (71.0%)	736 (70.9 %)

NB: Where numbers do not add up to full cohort, values are missing. Percentages refer to the data available

^a^ GOLD classification severity based on airflow limitation post-bronchodilator (*FEV1* forced expiratory volume during the first second) based on the European Community of Coal and Steel (*ECCS*) equations, *SD* standard deviation, *CAT* COPD Assessment Test, *GOLD* Global Initiative for Chronic Obstructive Lung Disease, *GCSE* General Certificate of Secondary Education

Despite being on the COPD register, 128 respondents (12.7%) did not meet the NICE criteria for airflow obstruction at the baseline study assessment. Of those that did, most respondents (701; 67.4%) had mild to moderate disease (GOLD stage 1 and 2). Almost two thirds (62.9%) of respondents reported severe dyspnoea (Medical Research Council (MRC) grade 3–5) and the mean COPD Assessment Test (CAT) score was 19.3 (SD 8.6), indicating moderate impact on patients’ lives. The most prevalent co-morbidities reported by patients were hypertension (481; 45.4%) and asthma (408; 38.5%). There were no marked differences in baseline characteristics between all included participants diagnosed with COPD and respondents to the 6-month questionnaire (Table 1).

### Identifying self-management behaviours

The majority of respondents (940; 94.0%) reported taking their inhalers or medications as instructed (Table 2). In the preceding 6 months, most reported no change in smoking habit from baseline (743; 85.5%), although 105 smokers (12.1%) reported quitting and 21 (2.4%) reported taking up smoking. Overall, 89.2% (962) of patients reported receiving their annual vaccinations, which was comparable between those under or over 65 years old. Most participants reported some form of physical exercise in the last seven days: walking (36.0%), moderate exercise (19.8%) and, vigorous exercise (14.2%), although participants reported sitting for an average of 5 h per day.

**Table 2:** Self-management behaviours: adherence to medication and lifestyles

Characteristic	
Adherence to medication	
Taking inhalers or medicines exactly as instructed by health professional; *n* (%)	
Yes	940 (94.0%)
No	60 (6.0 %)
Smoking habit at 6-months; *n* (%)	
No change since baseline	743 (85.5%)
Current smoking	193 (22.2%)
Former smoker	450 (51.8%)
Never smoker	100 (11.5%)
Change since baseline	126 (14.5%)
Quit smoking	105 (12.1%)
Started/resumed smoking	21 (2.4%)
Influenza vaccination usually received	
Total; *n* (%)	962 (89.2%)
Those under 65 years old; *n*	297
Receiving annual vaccination; *n* (%)	258 (86.9%)
Those over 65 years old; *n*	781
Receiving annual vaccination; *n* (%)	704 (90.1%)
Self-reported exercise in the last 7 days	
Vigorous^a^ exercise; *n* (%)	153 (14.2%)
Moderate^b^ exercise; *n* (%)	213 (19.8%)
Walking; *n* (%)	388 (36.0%)
Hours reported sitting; hours/day; median (IQR)	5 [4–8]

NB: Where numbers do not add up to full cohort, values are missing. Percentages refer to the data available

^a^ Heavy lifting, digging, aerobics or fast bicycling

^b^ Carrying light loads, bicycling at a regular pace or doubles tennis

### General COPD knowledge

The mean overall disease knowledge score of respondents on the Bristol COPD Knowledge Questionnaire (BCKQ) scale was 31.5 (SD 10.7) out of a maximum score of 65 (score range: 0–56). This corresponds to answering 48.4% of questions correctly (Table 3). Patients scored most highly on the five questions about the benefits of smoking cessation (mean 3.3 (SD 1.0) out of 5). Other topics where mean scores were above three included knowledge of recommendations on annual influenza vaccinations and causes of COPD such as smoking and occupational risks. Patients scored lowest on the questions regarding use, benefits and side effects of inhaled steroids (mean 0.7 (SD 1.0) out of 5). There were also low scores on the questions about oral steroids (mean 1.5 (SD 1.4) out of 5).

**Table 3:** Self-management behaviours: Bristol COPD Knowledge Questionnaire (BCKQ)

Topics of BCKQ^a^	Mean score (SD) (min = 0, max = 5)
1 General	2.1 (1.1)
Questions relating to chronicity, progression of disease over time, diagnosis, age of diagnosis and oxygen levels	
2 Aetiology	3.2 (1.4)
Questions relating to causes: smoking, dust, asthma, inheritance	
3 Symptoms	2.7 (1.4)
Questions relating to recognising common symptoms: e.g., fatigue, wheezing	
4 Breathlessness	2.3 (1.1)
Questions relating to breathlessness and to eating meals, oxygen levels and exercise	
5 Phlegm	2.7 (1.4)
Questions relating to sputum and dehydration, bronchodilators and breathing exercises	
6 Infections	2.2 (1.3)
Questions relating to signs of infections: sputum colour change, temperature, steroid use in exacerbation	
7 Exercise	2.9 (1.5)
Questions relating to benefits of exercise and fitness, bone density, depression and breathlessness	
8 Smoking	3.3 (1.0)
Questions relating to smoking cessation and risk of heart disease, further lung damage, lung function and NRT	
9 Vaccination	3.1 (1.0)
Questions relating to recommendations of annual influenza vaccination, age and protective factors	
10 Inhaled bronchodilators	1.8 (1.4)
Questions relating to use of bronchodilators, spacer devices and side effects.	
11 Antibiotics	2.3 (1.4)
Questions relating to use, side effects, effectiveness of antibiotics and exacerbations	
12 Oral steroids	1.5 (1.4)
Questions relating to steroid use, infection and side effects	
13 Inhaled steroids	0.7 (1.0)
Questions relating to use with oral steroids, spacer devices, effectiveness and side effects	
Overall BCKQ score (mean (SD))	31.5 (10.7)
Distribution of BCKQ (*n* (%))	
0–13	37 (5.8%)
14–26	150 (23.7%)
27–39	295 (46.5%)
40–52	144 (22.7%)
53–65	8 (1.3%)

*NRT* nicotine replacement therapy

^a^ Each of the 13 topics contained five questions (1 point for a correct answer). Total BCKQ score is out of 65 (13 topics × 5 questions)

### Exacerbation-related knowledge

Table 4 describes patients’ knowledge of how to recognise and manage exacerbations. Patients who had experienced an exacerbation within the last 6 months tended to be more likely to give the correct answer. In comparison to patients who had not reported an exacerbation in the last 6 months, those reporting an exacerbation were more likely to have both antibiotics (45.1% vs. 17.8%, *p* < 0.05) and steroids (40.1% vs. 14.1%, *p* < 0.05) at home. Irrespective of prior exacerbation, most patients were confident/very confident in using both antibiotics and steroids, and reported knowing what to do (e.g., adjust bronchodilator therapy or when to call an ambulance) if their breathing deteriorated.

**Table 4:** Self-management behaviours: exacerbation-related symptom recognition and treatments at home

	Characteristic	No exacerbation within the last 6-months (*n* = 534)	Exacerbation within the last 6-months (*n* = 490)	
Recognition of symptoms of exacerbation (question from BCKQ)	In depth breakdown of question 6 from BCKQ			
	With exacerbations phlegm usually becomes coloured (yellow or green); *n* (%)			
	Correctly answered	436 (85.7%)	435 (91.0%)	
	Incorrectly answered	10 (1.9%)	12 (2.5%)	
	Did not know	63 (12.4%)	31 (6.5%)	
	Exacerbations can occur in the absence of a chest infection; *n* (%)			
	Correctly answered	160 (32.3%)	180 (38.6%)	
	Incorrectly answered	53 (10.7%)	55 (11.8%)	
	Did not know	283 (57.1%)	231 (49.6%)	
	Chest infections are always accompanied by a high temperature; *n* (%)			
	Correctly answered	100 (20.0%)	105 (21.6%)	
	Incorrectly answered	185 (37.1%)	207 (42.6%)	
	Did not know	214 (42.9%)	152 (31.3%)	
Antibiotics	Course of antibiotics at home if needed; *n* (%)	91 (17.8%)	216 (45.1%)	
	Confidence in using at home antibiotics^a^; *n* (%)			
	Very confident	44 (48.4%)	106 (49.1%)	
	Confident	38 (41.8%)	96 (44.4%)	
	Not confident	6 (6.6%)	9 (4.2%)	
	Not at all confident	0	0	
Steroids	Course of steroids at home if needed; *n* (%)	71 (14.1%)	189 (40.1%)	
	Confidence in using steroid course at home^b^; *n* (%)			
	Very confident	36 (50.7%)	91 (48.1%)	
	Confident	29 (40.9%)	76 (40.2%)	
	Not confident	5 (7.0%)	14 (7.4%)	
	Not at all confident	0	4 (2.1%)	
Deterioration	What to do if breathing gets worse; *n* (%) (e.g., take two inhaler puffs)			
	Told with written instructions	72 (14.8%)	104 (22.0%)	
	Told but not written	244 (50.0%)	256 (54.1%)	
	Not told but know	97 (19.9%)	80 (16.9%)	
	Not told and do not know what to do	75 (15.4%)	33 (7.0%)	
Emergency services	When to call ambulance if breathing worsens; *n* (%)			
	Told with written instructions	34 (7.0%)	53 (11.3%)	
	Told but not written	99 (20.3%)	145 (30.9%)	
	Not told but know	249 (51.0%)	196 (41.8%)	
	Not told and do not know what to do	106 (21.7%)	75 (16.0%)	

NB: Where numbers do not add up to full cohort, values are missing. Percentages refer to the data available

*BCKQ* Bristol COPD Knowledge Questionnaire

^a^ Of those with ‘at home’ antibiotics, the confidence in use

^b^ Of those with ‘at home’ steroids, the confidence in use

### Reporting self-management support

Four-hundred (40.4%) patients reported having an agreed self-management plan, including only 214 (44.9%) of those with exacerbations in the last 6 months (Table 5).

**Table 5:** Self-management support: Plans and healthcare professional advice

Characteristic	No exacerbation within the last 6-months (*n* = 534)	Exacerbation within the last 6-months (*n* = 490)
Agreed self-management plan for COPD with health professional; *n* (%)		
Yes (within last 12-months)	129 (25.1%)	165 (34.6%)
Yes (more than 12-months ago)	57 (11.1%)	49 (10.3%)
No	276 (53.8%)	214 (44.9%)
Do not know	51 (9.9%)	49 (10.3%)
Current satisfaction with information provided about inhalers or medicines (by health professional); *n* (%)		
Understand everything	421 (80.2%)	352 (80.7%)
Understand but would like to know more	80 (15.2%)	57 (13.1%)
Slightly confused about medicines	15 (2.9%)	23 (5.3%)
Very confused about medicines	9 (1.7%)	4 (0.9 %)
Health professional advised to give up smoking within the last 12 months; *n* (%)		
If current-smoker at baseline	141 (54.7%)	149 (57.8%)
Health professional offered practical^a^ help to give up smoking within last 12 months; *n* (%)		
If current-smoker at baseline	105 (40.7%)	115 (44.6%)
Health professional advice on diet/eating; *n* (%)		
None	291 (56.6%)	247 (52.1%)
Lose/gain weight	73 (14.4%)	73 (15.5%)
Eat healthy food	164 (32.3%)	162 (34.4%)
Eat several small meals/day	28 (5.5%)	57 (12.2%)
Health professional advice to do some physical activity; *n* (%)	258 (50.2%)	261 (55.4%)
Attendance at a training course on COPD (e.g., Expert Patients Programme); *n* (%)		
Within the last 12-months	26 (5.0%)	50 (10.6%)
Yes but not within the last 12-months	34 (6.6%)	59 (12.5%)
No	456 (88.4%)	363 (76.9%)
Attendance at a support group for lung problems (e.g., Breathe easy); *n* (%)		
Regularly	5 (1.0%)	26 (5.5%)
Occasionally	10 (1.9%)	24 (5.1%)
Never	503 (97.1%)	421 (89.4%)
Attendance at pulmonary rehabilitation; *n* (%)		
Offered pulmonary rehabilitation in past 6 months	19 (3.7%)	53 (11.5%)
If offered, attended pulmonary rehabilitation	13 (68.4%)	32 (60.4%)

NB: Where numbers do not add up to full cohort, values are missing. Percentages refer to the data available

^a^ Practical help: e.g., NRT/smoking cessation clinic

The majority of respondents (773; 80.4%) appeared satisfied with the information given to them by their healthcare professionals about their medications. Of the 258 respondents who were current smokers at baseline, 56.2% had been advised by a healthcare professional to give up smoking within the last 12 months, and 42.7% were offered practical help (e.g., nicotine replacement therapy; NRT) to do so. Approximately half of respondents reported ever having been advised about diet or exercise, but only 15.0% had been advised to lose or gain weight.

Reported attendance at training courses (7.7%) and support groups (3.1%) in the last 12 months was low. Of the 72 patients (6.7%) that had been offered pulmonary rehabilitation in the past 6 months, 45 (62.5%) had attended.

Table 6 shows the association between (1) having a self-management plan; (2) exacerbation in the last 6 months; and positive health behaviours, knowledge about COPD and quality of life.

**Table 6:** Knowledge, behaviours and advice associated with having a self-management plan and report of recent exacerbation

	Self-management plan in place^a^	Exacerbation in the last 6 months
Attendance at training course^b^		
OR^c^ (95% CI)	2.72 (1.81, 4.12)*	1.73 (1.13, 2.65)*
Attendance at support group^d^		
OR^c^ (95% CI)	6.28 (2.96, 13.35)*	2.47 (1.13, 5.38*)*
BCKQ score		
Mean difference^c^ (95% CI)	4.87 (3.16, 6.58)*	4.39 (2.62, 6.15)*
Offered NRT by healthcare professional^e^		
OR^c^(95% CI)	1.40 (0.83, 2.38)	0.90 (0.53, 1.55)
Adherence to medications^f^		
OR^c^ (95% CI)	3.10 (1.43, 6.72)*	2.73 (1.31, 5.69)*
Ever advised to carry out physical activity by health professional		
OR^c^ (95% CI)	2.44 (1.78, 3.35)*	1.08 (0.78, 1.49)
CAT score		
Mean difference^c^(95% CI)	−0.53 (−1.61, 0.56)	2.89 (1.75, 4.02)*

*p-value < 0.05

^a^ Self-management/personal care plan ever agreed with a health professional, i.e., <12 months or >12 months ago

^b^ Ever attended a training (e.g., Expert Patients Programme) course i.e., <12 months or >12 months ago

^c^ Adjusted for age, gender, smoking status, education, MRC dyspnoea score and co-morbidities

^d^ Ever attended a support (e.g., Breathe Easy) group i.e., regularly or occasionally

^e^ Health professional offering help to give up smoking (e.g., NRT or a referral to a smoking cessation clinic) within last 12 months regardless of smoking status

^f^ Adherence: trying to take inhalers or medicines exactly as instructed by a doctor or nurse

### Identifying behaviours associated with having self-management plans

After adjusting for age, sex, smoking status, educational level, MRC dyspnoea score and number of co-morbidities, those who had a self-management plan in place were more likely to have attended a training course (odds ratio (OR) 2.72; 95% confidence interval (CI) 1.81–4.12), support group (OR 6.28; 95% CI 2.96–13.35), or to have received advice on physical activity (OR 2.44; 95% CI 1.78–3.35). They were also more likely to have better COPD knowledge scores (mean difference 4.87; 95% CI 3.16–6.58), and self-reported adherence to medication (OR 3.10; 95% CI 1.43–6.72). These patients also tended to have better CAT scores and were more likely to have been offered NRT, although these effects were not statistically significant.

### Effect of exacerbations on self-management behaviour

Patients who experienced an exacerbation within the last 6 months were more likely to have ever attended a training course (OR 1.73; 95% CI 1.13–2.65) or a support group (OR 2.47; 95% CI 1.13–5.38), and have better COPD knowledge (mean difference 4.39; 95% CI 2.62–6.15) compared to those not reporting an exacerbation (Table 6). In addition, patients with a recent exacerbation were more likely to adhere to medication (OR 2.73; 95% CI 1.31–5.69).

## Discussion

### Main findings

In this study, we report self-management support and behaviour patterns among a large, broadly representative primary care cohort of COPD patients in England. Although the majority of participants reported taking their medications as instructed by their healthcare professional and receiving annual influenza injections, a striking finding is that less than half of respondents reported having an agreed self-management plan; including patients reporting a recent exacerbation. Similarly, despite 62.9% reporting severe dyspnoea and, therefore, having the potential to benefit from pulmonary rehabilitation,^15^ <7% were referred to the service. This may also explain the lack of self-management advice reported as it is a key component of pulmonary rehabilitation. Of note is that the majority of participants had no formal educational qualifications and over a quarter lived alone, which could have affected whether health professionals discussed or offered self-management plans.^16^ Furthermore, contrary to recommended guidance,^5^ less than half of patients with a recent exacerbation reported having antibiotics or steroids to take at home (rescue pack) if necessary.

Our findings indicate clear potential for improvement in the promotion of healthy lifestyles, considering that only 56% of current smokers reported receiving smoking cessation advice in the last year. In addition, only 15% were advised about weight management despite nearly three quarters of the sample being either overweight or obese and 2% being underweight.

It was interesting to note that participants’ general knowledge about COPD (BCKQ) was moderate, and improvement here has potential. Despite patients’ poor knowledge regarding the use and benefit of inhaled medications, the high proportion of patients reporting satisfaction with the level of information provided about their medication suggested a discrepancy between perceived and actual knowledge. Attempts to improve the content or mode of delivery of medication information may, therefore, be warranted to optimise patient knowledge of this important area.

Our study indicates various patient benefits associated (though not necessarily causal) with receiving a self-management plan, including increased medication adherence, greater disease knowledge, increased attendance of training courses and support groups, and improved quality of life. However, despite such benefits, presence of a self-management plan was not associated with practical support, such as NRT, being offered.

### Interpretation of findings in relation to previously published work

Self-management in COPD is a relatively under-researched topic in comparison with other chronic diseases.^17^ There is little other evidence regarding the reality of patients’ self-management behaviour or the support offered by healthcare professionals in the UK.

Our findings that patients adhere well and report understanding their medication reflect those of previous Canadian studies.^18–20^ However, in contrast to our study, a cross-sectional study^20^ found that community-dwelling COPD patients in Canada reported better engagement with other self-management behaviours, where higher proportions of patients engaged in aerobic activity (74% vs. our 14%) and breathing exercises (70%). They also reported that 68% of patients made collaborative management decisions with their healthcare professional and 20% had attended pulmonary rehabilitation.

Although the limited use of self-management plans has previously been reported in two small studies of patients with recent exacerbations in the UK^14^ and Canada,^19^ our study supports and extends these observations in a larger generalisable primary care population. Furthermore our study adds to this by indicating that, in the UK, self-management plans are also associated with better reported adherence to medication, more support/self-management advice and better quality of life.

### Strengths and limitations of this study

We have described major facets within self-management behaviours in a large COPD population, from general practices representative of primary care in the UK.

It is, however, possible that the relatively low recruitment rate into the cohort (24%) might affect generalisability to a certain extent as participants were more likely to be male and of white British ethnicity than all eligible COPD patients in our participating general practices.^21^

While we have demonstrated that presence of a self-management plan is associated with better support and behaviours, the analyses were conducted using cross-sectional data. Analyses would need to be repeated with longitudinal data to ascertain whether the association was causal, or simply indicated that patients received a better overall package of care from their practitioner.

Study questionnaires completed by patients in the current analysis did not explore patients’ self-efficacy and motivation^17, 22^ in relation to self-management behaviour. Mediating factors, such as willingness to quit in the case of smoking cessation, would need to be accounted for in analyses to assess the true impact of self-management plans. Inconsistencies in the definition and measurement of self-management^7^ further limit our ability to compare the findings with existing evidence. The date of COPD diagnosis was not available for this analysis; hence we were unable to explore if duration of disease impacted on self-management behaviour or support.

Data presented in this paper were collected via patient self-completed questionnaire as objective measures were not feasible, although validated instruments were used where possible. As a result, data should, therefore, be interpreted with appropriate caution, allowing for potential biases. Self-report of behaviours such as compliance with medication is likely to be prone to overestimation by respondents compared with objective measurement.^23^ However, it is unclear whether the relationship between having a self-management plan and adherence to medication would be affected. Additionally, the BCKQ COPD knowledge questionnaire is now dated, and question 13 regarding knowledge about inhaled steroids is no longer current. However, this only represents 5 out of a total of 65 points and the questions about inhaled bronchodilators also scored low marks. We also asked questions regarding the confidence of patients to act (such as to start taking their rescue medication) during their exacerbation. Confidence rates were high, but it is acknowledged that such positive self-efficacy does not always translate into timely action.^24^ We were not able to observe actual self-administration of medication during exacerbations.

### Implications for future research, policy and practice

Despite NICE recommendations that all COPD patients should receive an individualised self-management plan,^5, 6^ implementation in practice appears clearly inadequate. Understanding this mismatch, particularly from the healthcare practitioner perspective, is an important area for future research in order to understand the potential barriers and enablers involved. Annual reviews of COPD patients in primary care seem the ideal opportunity to discuss self-management, as they are attended by over 90% of patients^2^ and already include some relevant aspects, e.g., desire to stop smoking, inhaler technique and referral to further services such as pulmonary rehabilitation. Wider health promotion behaviours such as weight management and physical activity were apparently not discussed by healthcare professionals, and had low uptake among patients. Improving the general health of patients could benefit management of their COPD symptoms, such as breathlessness, and improve outcomes.^25^

The time constraint within primary care consultations is a potential barrier to the inclusion of thorough self-management support,^25^ with an alternative being referrals to allied services. However, the majority of patients (especially those without self-management plans) had never attended a training course, a support group or pulmonary rehabilitation, indicating a lack of self-management support outside primary care-based patient and healthcare professional interactions. Further research is needed to explore the adequacy of referral pathways in primary care, whether resources are available to support training courses and pulmonary rehabilitation, as well as patients’ reasons for poor participation in existing services.^26^

Future implementation research^27^ is needed to explore how to expand current self-management recommendations to all COPD patients, within the confines of current financial constraints.

Finally, engaging the patient in their condition is a major challenge for health professionals managing COPD and other long-term health conditions, which if achieved could improve patients’ self-management behaviour. Studies report compliance rates of approximately 40% with self-management advice among patients with COPD,^28, 29^ with those who successfully self-manage their condition being younger and not living alone. Recent evidence suggests that motivation of non-compliant patients may be enhanced through multimodal approaches (e.g., health coaching, counselling, pulmonary rehabilitation), reporting reductions in readmissions and improved quality of life.^30^

## Conclusions

This study increases understanding about the real life practice of self-management behaviours in COPD patients. Patients are indeed partially using a self-management approach in their condition as recommended by bodies such as NICE. Adults with COPD are using inhaled medications, having annual influenza vaccinations, and some are engaging in partnerships with the healthcare service. However, healthcare professionals need to make explicit self-management plans with all of their patients, provide consistent advice and offer practical support regarding smoking cessation, diet, exercise and attendance at support groups, and actively ensure that their patients understand their condition and its management. This may require a new approach in primary care to achieve satisfactory implementation.

## Methods

### Study design

Cross-sectional analysis of data collected as part of the Birmingham COPD Cohort Study between 2011 and 2015, to establish self-management behaviours and support; and outcomes associated with having a self-management plan among primary care patients with COPD.

### Setting and participants

Recruitment to the Birmingham COPD Cohort Study has been described elsewhere.^21^ The cohort comprises patients with existing diagnosed COPD, newly case-found COPD and those with chronic respiratory symptoms without airflow obstruction. This analysis focuses on those with existing diagnosed COPD. Briefly, 6383 patients aged 40 years or over with a GP-recorded COPD diagnosis from 71 general practices across the West Midlands, UK, were invited to take part, of whom 1558 (24.4%) were eventually consented into the study. At the time of this analysis, 1547 had consented. The current analysis was based on prevalent patients with 6-month questionnaire data (*n* = 1078), which specifically asked about self-management behaviours.

### Data collection

Participants attended a baseline study assessment at their own GP practice, conducted by trained research assistants. Patients completed questionnaires at this assessment and then at 6-monthly intervals, with a further final face-to-face clinical assessment at approximately three years. The questionnaires were self-completed and included items on sociodemographic characteristics, health (including health related quality of life and co-morbidities), lifestyle behaviours, medications, health service usage and disease knowledge. Validated tools were used where possible. Data for the current analyses came primarily from the 6-month questionnaire or baseline assessment as appropriate.

### Data and variables

At baseline assessment, trained research assistants performed spirometry using the nddEasy One spirometer (ndd, Switzerland), pre and post administration of 400 µg salbutamol. A minimum of three and a maximum of six blows post-bronchodilator were allowed, until repeatability within 100 mls was reached and the best reading was recorded. Specialised software (MMiller) and real-time show of volume-time and flow-volume graphs were used to ensure quality. All traces were over-read and data for forced expiratory volume in 1 s (FEV_1_) and forced vital capacity (FVC) were deemed viable if they met American Thoracic Society acceptability criteria and were repeatable to within 200 ml. COPD was defined as airflow obstruction using a fixed ratio of FEV_1_/FVC < 0.70 and severity was graded according to the European Community of Coal and Steel (ECCS) equations.^31^

We included several variables describing self-management knowledge and behaviour in the 6-month questionnaire (Supplementary information 1). The BCKQ has 13 main themes each with five sub questions, and measures patients’ general knowledge about COPD (higher scores indicating higher knowledge).^32^ Physical activity levels were measured using the International Physical Activity Questionnaire.^33^ We also asked patients to report whether they took medications as prescribed, if they had a course of antibiotics and steroids at home to take in the event of an exacerbation and confidence in their use, their smoking habit and receipt of influenza vaccinations.

Variables describing self-management support included: having an agreed self-management plan with a health professional, satisfaction with information on how to take medications and inhalers, advice from healthcare professionals on nutrition, physical activity and smoking cessation. We also asked about attendance at training or support courses on COPD and pulmonary rehabilitation.

Disease specific quality of life was assessed using the CAT.^34^ The MRC dyspnoea score was used to assess degree of breathlessness.^35^ Co-morbidities of interest included cancer, diabetes, hypertension, coronary heart disease, heart failure, asthma and depression. Exacerbations were defined as the report of one or more courses of either steroids or antibiotics taken within the time period.

### Statistical analyses

All analyses were undertaken in Stata13.^36^ The characteristics of the study population, self-management behaviour and support were described using simple descriptive statistics. Analyses were stratified by the experience of an exacerbation within the last 6 months to determine whether this affected symptom recognition, available treatments at home and self-management support provided by healthcare professionals. Multiple regression models were used to investigate the relationship between (1) having a self-management plan, (2) having an exacerbation within the previous 6 months, and reported self-management behaviours and support, adjusting for age, sex, smoking status, education, MRC dyspnoea score and co-morbidities.

### Ethics

The cohort received ethical approval from the National Research Ethics Service Committee West Midlands, Solihull (ref.: 11/WM/0304). Methods were performed in accordance with relevant regulations and guidelines. Written informed consent was obtained from each participant at the start of the baseline assessment.

### Data availability

The data that support the findings of this study are held by the BLISS research team at the University of Birmingham. Copies of the questionnaires, measurement procedures and administrative processes are available on request, through our website [www.birmingham.ac.uk/bliss]. The data are not publicly available due to them containing information that could compromise research participant privacy/consent.

## Electronic supplementary material

Appendix 1
