# Supplementary material for: Self-management behaviour and support among primary care COPD patients: cross-sectional analysis of data from the Birmingham Chronic Obstructive Pulmonary Disease Cohort
Source: NPJ Prim Care Respir Med. 2017 Jul 20;27:46. doi: 10.1038/s41533-017-0046-6 (PMC5519687; doi:10.1038/s41533-017-0046-6)
Supplement: Supplementary file 1 — Appendix 1 [file 41533_2017_46_MOESM1_ESM.docx]

**Appendix 1: Variables from six-month questionnaire**

| **Variable** | **Original question as scripted in questionnaire** | **Validated tool** |
| --- | --- | --- |
| Taking medications | Do you try to take your inhalers or medicines exactly as you have been instructed by a doctor or nurse? |  |
| Change in smoking habit | Have there been any changes to your smoking habits in the last six months? |  |
| Influenza vaccination | Do you usually receive the flu jab in the winter? |  |
| Exercise reporting | How much time did you usually spend doing vigorous/moderate/walking physical activities on one of those days?  During the last 7 days, how much time did you spend sitting on a week day? | IPAQ |
| Disease specific knowledge | BRISTOL COPD KNOWLEDGE QUESTIONNAIRE (BCKQ)© | BCKQ |
| Antibiotic course at home | Do you have a course of antibiotics at home to use if needed? |  |
| Confidence in using antibiotics | If yes, please state how confident you feel about when you should take the medication yourself? |  |
| Steroid course at home | Do you have a course of steroids at home to use if needed? |  |
| Confidence in using steroids | If yes, please state how confident you feel about when you should take the medication yourself? |  |
| Breathing deterioration | What sentence best describes what you have been told to do if your breathing gets worse (e.g. take 2 puffs instead of one)? |  |
| Emergency Services | Have you been told when you should call an ambulance if your breathing worsens? |  |
| Self-management plan | In the last 12 months, have you and a health professional agreed a personal care plan/self-management plan for COPD? |  |
| Satisfaction about medication | Are you satisfied with the information doctors and nurses have given you about your inhalers or medicines? |  |
| Health professional advice on smoking cessation | In the last 12 months, has a doctor or nurse advised you to give up smoking? |  |
| Health professional offered practical help to give up smoking | In the last 12 months, has a doctor or nurse offered to help you to give up smoking (e.g. given you nicotine gum or patches or a referral to a smoking cessation clinic)? |  |
| Health professional advice on diet | What have doctors or nurses told you about your diet or eating? |  |
| Health professional advice on physical activity | Have you been told by a doctor or nurse to try to do some physical activity (e.g. walking, brisk walking and other forms of exercise)? |  |
| Attendance at a training course | Have you attended a training course on your condition e.g. Expert Patients Programme? |  |
| Attendance at a support group | Do you ever attend a support group for your lung problems e.g. Breathe Easy? |  |
| Offer of pulmonary rehabilitation | In the last six months, have you been offered pulmonary rehabilitation? |  |
| Attendance at pulmonary rehabilitation | If yes, have you attended pulmonary rehabilitation? |  |
